# Trials of Life

**Published:** 1861-05

**Authors:** 


**TRIALS OF LIFE.**—We start upon life's journey full of hope, full of gladness, and full of joyous ambition, confident in our own strength and in the support of friends and kindred stationed round about us, on whom we lean with great satisfaction; but as years pass on, one of the outposts, the supports, falls; and then another and another, each succeeding year, leaving one or more the less. For a while we scarcely miss the acquaintances and friends of our childhood, for we have so many; but as time rolls on, the number becomes so small that each additional loss makes a greater void. Father, mother, brothers, sisters, our oldest neighbors, all gone; the minister of our youth has grown gray before us—he, too, has passed away; and beyond a schoolmate here and another there, nothing is left to connect us with the times and the home of childhood, and such a feeling of desolation comes over us, that we are ready to sink in perfect helplessness and despair. To the old who may chance to read these lines, the suggestion is made, which, if wisely heeded, may save the body from sinking under the whelming load, and it is this, He who made us, is the Father of us all; and the dispensations of this life are designed to prepare us the more certainly for a beatific existence beyond the grave, and to enable us to make the transition with the least violence, and at the same time to train us to those habitudes of heart which will the more elevate us in the world beyond, he arranges that we shall learn to lean less on ourselves, less on others, and more on Himself, as a weary man leans on a staff; and the sooner we begin to learn thus to lean, the happier we shall be in time, and the more ready shall we find ourselves to take up the returnless journey without a murmur and without a sigh.

There are no words more beautiful and more true in any language than that "GOD IS LOVE" to all his true children; and the longer they live the more constantly does he gather himself about them with his providences, not certainly in the way that man's wisdom would devise, but in the manner most surely to eventuate in their safe arrival at their heavenly home. So that, while it is natural that we should feel the death of those who are near to us more and more acutely, the older we grow, we should gain even physical power to resist the most crushing trials, in the sweet reflection that, behind the darkest cloud, a loving Father hides a face all radiant with pity, sympathy, and affection, to be shown in due time when faith has done its perfect work. So that, for life's sufferings, there is a balm in Gilead, there is a Physician there!

